# A panel of DNA methylation biomarkers for detection and improving diagnostic efficiency of lung cancer

**DOI:** 10.1038/s41598-021-96242-6

**Published:** 2021-08-18

**Authors:** Bing Wei, Fengxin Wu, Wenqun Xing, Haibo Sun, Chi Yan, Chengzhi Zhao, Dongqing Wang, Xiaobing Chen, Yanli Chen, Mingming Li, Jie Ma

**Affiliations:** 1 Department of Molecular Pathology, Affiliated Cancer Hospital of Zhengzhou University, Henan Cancer Hospital, No 127, Dongming Road, Zhengzhou, 450008 Henan China; 2 Henan Key Laboratory of Molecular Pathology, Zhengzhou, Henan China; 3 Department of Thoracic Surgery, Affiliated Cancer Hospital of Zhengzhou University, Henan Cancer Hospital, Zhengzhou, Henan China; 4 Excellen Medical Technology Co., Ltd., Beijing, China

**Keywords:** Lung cancer, Tumour biomarkers, Diagnostic markers, DNA methylation

## Abstract

Lung cancer remains the leading cause of cancer deaths worldwide. Although low-dose spiral computed tomography (LDCT) screening is used for the detection of lung cancer in a high-risk population, false-positive results of LDCT remain a clinical problem. Here, we developed a blood test of a novel panel of three established lung cancer methylation biomarkers for lung cancer detection. Short stature homeobox 2 gene (*SHOX2*), ras association domain family 1A gene (*RASSF1A*), and prostaglandin E receptor 4 gene (*PTGER4*) methylation was analyzed in a training cohort of 351 individuals (197 controls, 154 cases) and validated from an independent cohort of 149 subjects (89 controls, 60 cases). The novel panel biomarkers distinguished between malignant and benign lung disease at high sensitivity and specificity: 87.0% sensitivity [95% CI 80.2–91.5%], 98.0% specificity [95% CI 94.9–99.4%]. Sensitivity in adenocarcinoma, squamous cell carcinoma, small cell lung cancer, and other lung cancer was 89.0%, 87.5%, 85.7%, and 77.8%, respectively. Notably, cancer patients in stage I and II showed high diagnostic sensitivity at 82.5% and 90.5%, respectively. Moreover, the diagnostic efficiency did not show bias toward age, gender, smoking, and the presence of other (nonlung) cancers. The performance of the panel in the validation cohort confirmed the diagnostic value. These findings clearly showed that this panel of DNA methylation biomarkers was effective in detecting lung cancer noninvasively and may provide clinical utility in stand-alone or in combination with current imaging techniques to improve the diagnosis of lung cancer.

## Introduction

Lung cancer is the second most common cancer and the leading cause of cancer deaths worldwide^1,2^. It surpassed breast cancer as the highest cause of cancer-related deaths in women. Lung cancer is expected to account for 22% of all female cancer deaths and 22% of all male cancer deaths in 2021^2^.

Current detection methods of lung cancer include the testing of sputum, x-ray, and computed tomography (CT) scanning of the chest. Studies have shown that using chest x-ray and sputum cytology as screening techniques does not reduce the mortality rate of lung cancer^3,4^. Low-dose spiral computed tomography (LDCT) has been demonstrated to be more sensitive than chest radiography in high-risk smokers for the detection of early-stage lung cancer. It has been shown to reduce the mortality rate^5,6^. However, the diagnostic accuracy of LDCT screening is limited due to its low specificity causing high downstream healthcare costs^5^. Therefore, novel noninvasive methods are urgently needed to improve lung cancer detection and reduce the mortality rate.

Molecular biomarkers might have the potential for improving the detection and management of lung cancer in the clinical setting^7,8^. Several biomarkers in gene expression^9^, somatic mutations^10^, copy number variations^11^, differences in methylation^12^, or the abundance of plasma proteins^13^ have been extensively studied for clinical research. In recent years, aberrant DNA methylation has been observed in various cancers and is considered to play a significant role in carcinogenesis^14,15^. DNA methylation is a relatively stable biochemical modification carried out by DNA methyltransferases. Methylation biomarkers can be detected not only from tissue but also in serum, fecal, and plasma^16^, indicating its potential as a noninvasive method for cancer detection. It has been reported that in vitro diagnostic tests using DNA methylation-based biomarkers have been approved by the U.S. Food and Drug Administration^17,18^.

Short stature homeobox gene two (*SHOX2*), ras association domain family 1A (*RASSF1A*), and prostaglandin E receptor 4 gene (*PTGER4*) methylation has been separately reported to be highly correlated with lung cancer diagnosis and prognosis. The promoter methylation of *SHOX2* has been identified as a valuable biomarker for lung cancer diagnosis in several research studies^19,20^. Promotor hypermethylation of *RASSF1A* frequently occurs in lung cancer and is frequently found in small cell lung cancer^21,22^. Methylated prostaglandin E2 receptor EP4 subtype (*mPTGER4*) was recently reported as a methylation marker for the differential diagnosis of benign and malignant lung diseases^23^. However, the combined detection of *SHOX2*, *RASSF1A,* and *PTGER4* methylation in plasma has hardly been reported. Extensive prospective screening trials have been validated that multiple gene panels could increase sensitivity and improve cancer diagnostic efficiency^17,18,24^.

In the presented study, a panel of DNA methylation of *SHOX2*, *RASSF1A*, and *PTGER4* was tested in blood plasma based on Real-Time PCR to distinguish between malignant lung cancer and controls (healthy individuals, benign lung disease, and patients with other cancer).

## Materials and methods

### Ethics

The participants were enrolled in Henan Cancer Hospital, the Affiliated Cancer Hospital of Zhengzhou University. All subjects signed the informed consent before blood collection, and they were informed of the usage of plasma and the test results. The study has been approved by the Medical Ethics Committee of Henan Cancer Hospital (2,018,157) and all methods were carried out in accordance with the relevant guidelines and regulations, as well as the Declaration of Helsinki.

### Study design, subjects, and computed tomography

The clinical study was designed and implemented in Henan Cancer Hospital using the Diagnostic Kit for Lung Cancer Genes Methylation (Excellen Medical Technology Co., Ltd.). Clinical status was not determined before blood draw for methylated genes assay, and blood samples were obtained from all subjects who met the selection criteria. All technicians were blinded to the clinical information of subjects.

**Figure 1:**
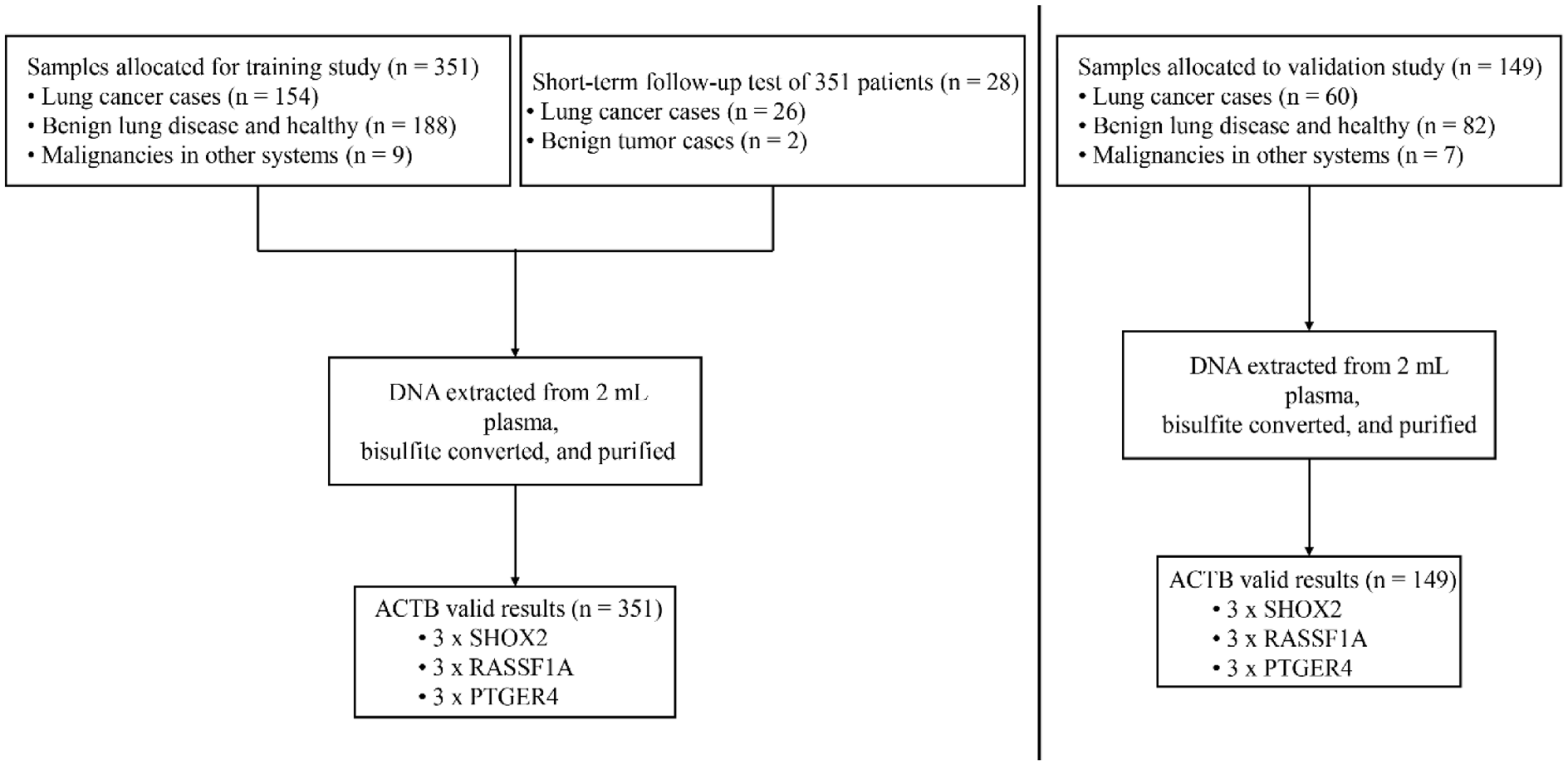
Sample disposition, study setup, and polymerase chain reaction (PCR) assay formats. Boxes in the bottom line indicate number of valid results and number of PCR replicates per PCR assay. For more details, see materials and methods section. *ACTB* Actin, beta gene, *SHOX2* Short stature homeobox 2 gene, *RASSF1A* Ras association domain family 1A gene, *PTGER4* Prostaglandin E receptor 4 gene.

Altogether, samples from 500 subjects passed the sample quality control acceptance criterion and were enrolled for analyzing the DNA methylation. The subjects were divided into a training set and a validation set by enrollment time. The initial series of 351 cases and controls were used for training and the subsequent series of 149 was used for validation. Figure 1 showed details of the sample disposition. Among 351 subjects of training studies, 154 were diagnosed with lung cancer (LC), including 82 adenocarcinomas, 40 squamous cell carcinomas, 14 small cell lung cancer, and 18 unclassified lung cancer patients. The other 197 cases were controls, including benign lung diseases such as pulmonary fibrosis, infection, sarcoidosis, and bronchiectasis, etc. Nine patients were diagnosed with malignancies in other systems, including one thyroid carcinoma, one osteosarcoma, and seven esophageal cancer (Table 1). The median age for LC cases and controls was 60 and 53 years, respectively. Subjects with lung cancer smoked more than controls (40 vs. 20 pack-years). The validation study was conducted with 20 healthy controls, 62 patients with nonmalignant lung disease, 7 patients with malignancies in other systems, and 60 patients with LC. More details of the subjects’ characteristics for the study were described in Table 1. The classification of all conditions was based on diagnosis from computed tomography and subsequent pathological examinations. LC patients were divided into four subgroups based on the TNM guidelines classification criteria^25^. These LC cases covered all major histological types and a broad range of stages.

**Table 1:** Subject’s demographics and clinical characteristics.

Subject characteristics	Training set (N = 351)		Validation set (N = 149)	
	Cases (n = 154)	Controls (n = 197)	Cases (n = 60)	Controls (n = 89)
**Age (years)**				
≤ 50 Years	24 (15.6)	90 (45.7)	10 (16.7)	23 (25.8)
51–60	56 (36.4)	53 (26.9)	22 (36.7)	37 (41.6)
61–70	62 (40.3)	46 (23.4)	26 (43.3)	26 (29.2)
> 70	12 (7.8)	8 (4.1)	2 (3.3)	3 (3.4)
Median age	60	53	60	57
Age range	36–78	17–76	26–78	20–73
**Gender**				
Male	102 (66.2)	126 (64.0)	46 (76.7)	56 (62.9)
Female	52 (33.8)	71 (36.0)	14 (23.3)	33 (37.1)
**Smoking status**				
Nonsmoker	71 (46.1)	175 (88.8)	25 (41.7)	76 (85.4)
Ex-smoker	29 (18.8)	8 (4.1)	14 (23.3)	4 (4.5)
Current smoker	54 (35.1)	14 (7.1)	21(35.0)	9 (10.1)
**Smoking pack years**				
Range packs/years	0–120	0–60	0–120	0–70
Median packs/years	40	20	40	30
Mean packs/years	41	21	42	23
**Histology subtype**				
Adenocarcinoma	82 (53.2)	–	27 (45.0)	–
Squamous cell carcinoma	40 (26.0)	–	18 (30.0)	–
Small cell lung cancer	14 (9.1)	–	7 (11.7)	–
Other	18(11.7)	–	8 (13.3)	–
healthy	–	43 (21.8)	–	20 (22.5)
Benign lung disease	–	145 (73.6)	–	62 (69.7)
Malignancies in other systems	–	9 (4.6)	–	7 (7.8)
**Stage**				
I	40 (26.0)	–	12 (20.0)	–
II	21 (13.6)	–	15 (25.0)	–
III	46 (29.9)	–	17 (28.3)	–
IV	44 (28.6)	–	15 (25.0)	–
Unknown	3 (1.9)	–	1 (1.7)	–

Benign lung diseases including pulmonary infection, sarcoidosis and bronchiectasis etc. Malignancies in other systems including thyroid carcinoma, breast cancer, osteosarcoma and esophageal cancer.

### Sample collection and storage

Samples were collected from outpatients and inpatients in Henan Cancer Hospital from January 2019 to December 2019, the sample information was recorded in sample collection forms. All subjects underwent a blood draw before the pathological examination, and subsequent biopsy or surgery was performed. None of the subjects received chemotherapy, radiotherapy, or surgical intervention before the blood draw and computed tomography. A 5 ml peripheral blood sample was collected with 5 ml K_2_EDTA anticoagulant tubes (BD biosciences, Franklin Lakes, NJ, USA) to ensure the assay's accuracy. Sample storage and transportation followed the instructions for the use of the Nucleic Acid Extraction Reagent (Excellen Medical Technology Co., Ltd.).

### DNA isolation and bisulfite conversion

Plasma was prepared within 4 h after the blood draw. After collecting the blood sample, it was centrifuged at 1500 g for 10 min; then, the supernatant was transferred to a 15 mL test tube and recentrifuged at 1500 g for 10 min. The plasma stored at -80 °C and avoided repeated freeze-thawing. DNA isolation from plasma and bisulfite conversion using the Nucleic Acid Extraction Reagent (Excellen Medical Technology Co., Ltd.) according to the respective instructions for use. Briefly, 3 mL Lysis Binding Buffer (main components of tris and guanidine isothiocyanate) were added to 2 ml plasma and incubated for 20 min at room temperature. Added 100 µl magnetic beads, and the mixture was incubated for 60 min at 10–20 rpm in a rotator. The reaction tube was placed into the Magnetic Racks for 1–3 min and discarded the supernatant. The beads were washed with Wash Buffer A [main components of tris and guanidine isothiocyanate and 50% (v/v) ethanol] and dried with the lid opened for 10 min at room temperature. Thereafter, the DNA was converted in a bisulfite reaction. 180 μl Bisulfite Solution (sodium bisulfite) and 20 μl Protection Buffer (tetrahydrofurfuryl alcohol) were added to the tube containing the magnetic beads. After vortexing magnetic beads completely, the microtube was placed into a thermoshaker and incubated for 45 min at 85℃ without shaking, which converts unmethylated cytosine residues to uracil residues. After the conversion reaction, 800 μl Lysis Binding Buffer was added and incubated for 30 min at room temperature in a thermomixer with 10–20 rpm. The tube was placed into Magnetic Racks, and the bound DNA was washed once with Wash Buffer A and three times with Wash Buffer B [15% (v/v) water and 85% (v/v) ethanol, absolute]. Finally, the bisulfite-converted DNA (bisDNA) was eluted in 35 µL and ready for use in real-time PCR.

### DNA methylation analysis

Bisulfite-modified DNA was used as a template for fluorescence-based Real-Time PCR according to the instructions of the Diagnostic Kit for Lung Cancer Genes Methylation (Excellen Medical Technology Co., Ltd.)^26^. In brief, amplification reactions were carried out in triplicate in a volume of 25 µL that contained 12.5 µL Reaction Buffer, 2.5 µL of Primer Mixture, and 10 µL of bisulfite-modified DNA. Amplifications were carried out using the following profile: 98 °C for 5 min, followed by 45 cycles at 95 °C for 10 s and 63 °C for 5 s to 58 °C for 30 s. Amplification reactions were carried out in 96-well plates in an Applied Biosystems 7500 Fast Real-Time PCR Systems (Applied Biosystems). The data was analyzed by Applied Biosystems 7500 Fast Real-Time PCR System Sequence Detection Software v1.4.1. Each plate included patient DNA samples, positive controls (in vitro methylated human DNA), negative controls (normal leukocyte DNA or DNA from a known unmethylated cell line), and multiple water blanks. CpGenome Universal Methylated DNA (S7821, Millipore) and Human Genomic DNA (G3041, Promega) were used as positive and negative controls, respectively.

In the PCR reaction, the primers and probes can discriminate between methylated and unmethylated DNA sequences, and the methylated sequences are preferentially amplified. Hydrolysis probes specific for the methylated target sequence used in the reaction can exclusively identify methylated target sequences during the PCR amplified reaction, and can be detected on fluorescence channels FAM, HEX, Texas Red and CY5. The PCR assay detected methylated *SHOX2*, *RASSF1A*, and *PTGER4* DNA as targets and *ACTB* DNA as internal control, to assess the adequacy of input DNA. This co-amplified internal control monitored the sample quality, preparation, and adequate DNA concentration of the sample. A sample showing no Ct or a value higher than 35.0 was considered invalid; as such high values were associated with very low bisDNA content or PCR inhibition. Five (1%) of the tests in the study failed to yield a result, due to insufficient amount of DNA in the sample. The CpG island track and qPCR target regions within the genome (*SHOX2*: chromosome 3:158,103,514–158,103,606, *RASSF1A*: chromosome 3:50,340,809–50,340,892, and *PTGER4*: chromosome 5:40,681,382–40,681,493; GRCh38/hg38) were showed in Supplementary Fig. S1. The sequences of primers and probes used in this study were shown in Supplementary Table S1.

Real-time PCR data were analyzed using the Lung Cancer Analysis software v2.2 (Excellen Medical Technology Co., Ltd.)^26^. The software calculated a composite score based on the cycle threshold (Ct) values of the three methylated genes through logistic regression. In the validation study, analysis was conducted by applying the trained model to the validation data. We obtained the optimal cutoff value in the training test at the maximum Youden index and evaluated the diagnostic efficiency of the test in the validation study using sensitivity and specificity. The final result was positive if the composite score is greater than or equal to the cutoff value, inverse negative if the score is less than the cutoff value. Analytical sensitivity of the assay was evaluated according to CLSI guidance documents (see online supplementary information).

### Statistical analysis

The performance of the assay was reported by using sensitivity and specificity. Sensitivity was defined as the ratio of correctly assigned positive lung cancer samples in all lung cancer samples. Specificity was defined as the ratio of correctly assigned negative samples in all normal/benign lung and other cancer samples. Positive predictive value (PPV; the probability that the disease is given a positive test result) and negative predictive value (NPV; the probability that the disease is absent given a negative test result) were also calculated. The receiver operating characteristic (ROC) and the area under the ROC curve (AUC) were analyzed using the IBM SPSS Statistics 24. The association of test positivity with demographic characteristics were assessed using the Chi-square test or Fisher exact test when less than 5 individuals were observed. Statistical significance was defined as *P* < 0.05.

## Results

### Receiver operating characteristic curve analysis and lung cancer diagnostic accuracy in training set

We tested quantitative analysis of promoter methylation in the plasma DNA samples from 154 lung cancer (LC) patients and 197 control subjects using a Diagnostic Kit for Lung Cancer Genes Methylation (Excellen Medical Technology Co., Ltd.) based on Real-Time PCR (RT-PCR). The LC cases covered all major histological types and a broad range of stages and are confirmed with pathological results. More detailed information of the population characteristics is described in methods. We performed receiver operating characteristic (ROC) curve analysis to analyze the diagnostic efficacy of the *SHOX2*, *RASSF1A* and, *PTGER4* methylation in blood plasma. As shown in Table 2, the methylation analysis of *SHOX2*, *RASSF1A*, and *PTGER4* panel showed a high diagnostic sensitivity of 87.0% and 98.0% specificity with an AUC value of 0.938. PPV and NPV are two other necessary measures of diagnostic accuracy. They are related to sensitivity and specificity through disease prevalence (Π). The PPV and NPV values were calculated with the formulas:

1 $${\text{PPV}} = \frac{Sensitivity*\Pi }{{Sensitivity*\Pi + \left( {1 - specificity} \right)*\left( {1 - \Pi } \right)}}$$

2 $${\text{NPV}} = \frac{{Specificity*\left( {1 - \Pi } \right)}}{{ \left( {Specificity *\left[ {1 {-}\Pi \left] { + } \right[1 {-} sensitivity} \right]*\Pi } \right)}}.$$

**Table 2:** The consistency of RT-PCR and pathological examination in detecting aberrant methylation of the *SHOX2*, *RASSF1A*, and *PTGER4* gene.

PCR (*SHOX2* + *RASSF1A* + *PTGER4*)	Pathological examination		Total
	Positive	Negative	
Positive	134	4	138
Negative	20	193	213
Total	154	197	351
AUC	0.938		
95%CI	0.907–0.961		
Kappa	0.86		
Sensitivity	87.0%		
Specificity	98.0%		
Positive predictive value (PPV)	51.7% (cancer prevalence of 2.4%)		
Negative predictive value (NPV)	99.7% (cancer prevalence of 2.4%)		

*SHOX2* Short stature homeobox 2 gene, *RASSF1A* Ras association domain family 1A gene, *PTGER4* Prostaglandin E receptor 4 gene, AUC Area under the curve.

In high-risk population (e.g., Lung cancer prevalence of 2.4%^27^), the PPV is then 51.7%. Correspondingly, the NPV is 99.7%. NPV and PPV values show that the *SHOX2*, *RASSF1A*, and *PTGER4* methylation detection of plasma could be an effective complementary tool in high-risk lung cancer diagnosis.

In addition, we also performed a short-term follow-up test on 28 subjects, of which 26 were lung cancer cases and two benign tumor cases. Blood methylation PCR was performed once before surgery (Included in Table 2) and was retaken five days after the operation (Not included in Table 2). The results showed that 26 cases of lung cancer samples were tested positive by PCR before the operation and negative after the operation. Two cases of benign tumor samples were detected negative before and after the operation. These results indicate that the three methylated gene markers of *SHOX2*, *RASSF1A*, and *PTGER4* originate from lung cancer tissue rather than from healthy tissue or benign tumors.

### Subtype analysis of *SHOX2*,* RASSF1A* and *PTGER4* methylation in plasma

**Figure 2:**
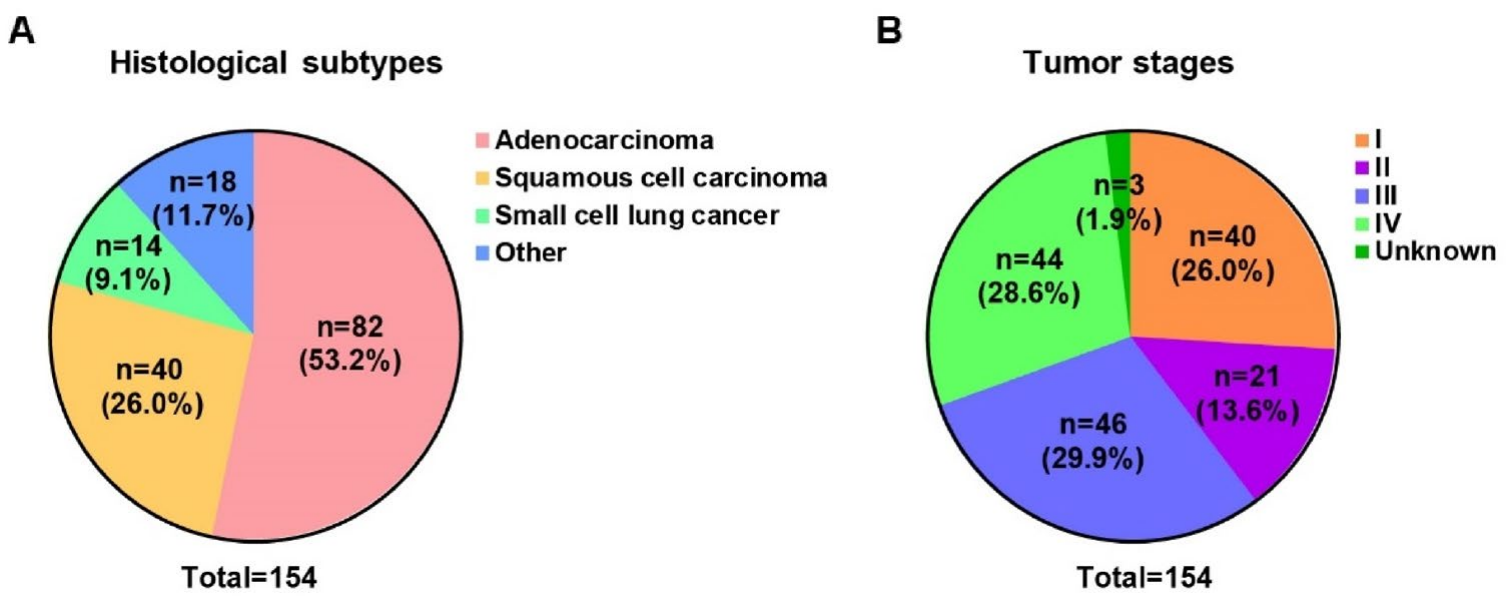
Histological subtypes (**A**) and tumor stages (**B**) of the patient population.

The performance of the *SHOX2*, *RASSF1A* and, *PTGER4* methylation in blood plasma was analyzed with respect to the histological subtypes, including adenocarcinoma, squamous cell carcinoma, small cell lung cancer, large cell carcinoma and other as shown in Fig. 2A and Table 3. The data showed that the sensitivity of the *SHOX2*, *RASSF1A* and *PTGER4* methylation panel in blood plasma was 87.0% in lung cancer group. In a more detailed analysis, the *SHOX2*, *RASSF1A* and, *PTGER4* methylation panel showed 89.0% in adenocarcinoma, 87.5% sensitivity in squamous cell carcinoma, 85.7% in small cell lung cancer and 77.8% sensitivity in other lung cancer, respectively, which means the *SHOX2*, *RASSF1A* and *PTGER4* methylation panel in plasma is a noninvasive biomarker with wide application in almost all histological subtypes of lung cancer. We further evaluated the sensitivity of the *SHOX2*, *RASSF1A*, and *PTGER4* methylation panel in plasma in different tumor stages (Fig. 2B and Table 3). The results showed that the methylation analysis of *SHOX2*, *RASSF1A*, and *PTGER4* in plasma showed high diagnostic ability with a sensitivity of 87.0%. Notably, the *SHOX2*, *RASSF1A*, and *PTGER4* methylation panel in plasma achieved a high sensitivity of 82.5% in stage I and 90.5% in stage II lung cancer patients, respectively.

**Table 3:** Subtype analysis.

Tumor stages	Histology				
	Adenocarcinoma	Squamous cell carcinoma	Small cell lung cancer	Other	All
I	28/33 (84.8%)	3/4 (75%)	0/0	2/3 (66.7%)	33/40 (82.5%)
II	12/14 (85.7%)	2/2 (100%)	1/1 (100%)	4/4 (100%)	19/21 (90.5%)
III	12/12 (100%)	21/23 (91.3%)	6/7 (85.7%)	2/4 (66.7%)	42/46 (91.3%)
IV	21/23 (91.3%)	8/10 (80%)	5/6 (83.3%)	4/5 (80.0%)	38/44 (86.4%)
Unknown	0/0	1/1 (100%)	0/0	2/2 (100%)	3/3 (100%)
All	73/82 (89.0%)	35/40 (87.5%)	12/14 (85.7%)	14/18 (77.8%)	134/154 (87.0%)

Stage and histology-specific performance of the *SHOX2*, *RASSF1A* and *PTGER4* methylation biomarkers using plasma samples from patients with suspected lung cancer.

### Overall performance of the panel in clinical subgroups

To identify possible biases in diverse demographic characteristics of the population, such as different ages, gender, and smoking history, an overall performance analysis of the panel was undertaken in the training set. Table 4 showed the details of this analysis. In age analysis, sensitivity was above 80% for most age groups, and specificity for LC was consistently high across all age groups. In gender analysis, sensitivity and specificity were similar among males and females, sensitivity/specificity of the lung cancer group was 86.3%/96.8% for males, compared with 88.5%/100% for females. The panel was performing well among different age and gender groups, no significant differences in the diagnostic efficiency were observed in relation to age and gender (χ^2^ test, *P* > 0.05). In addition, no significant sensitivity/specificity differences were observed among different smoking groups.

**Table 4:** Overall performance of the panel in diverse demographics characteristics of the population.

Clinical characteristics	Number of specimens	%		χ^2^ test
		Sensitivity	Specificity	*P* value
**Diagnosis**				
Lung cancer	154	87.0		
healthy	43		97.7	
Benign lung disease	145		98.6	
Other nonlung cancer	9		88.9	0.131
**Age**				
≤ 50 Years	114	79.2	98.9	
51–60	109	91.1	96.2	
61–70	108	85.5	97.8	
> 70	20	91.7	100	0.665
**Gender**				
Male	228	86.3	96.8	
Female	123	88.5	100	0.285
**Smoking status**				
None	246	87.3	98.3	
Former	37	86.2	100	
Current	68	87.0	92.9	0.082

### Validating the diagnostic efficiency of the three gene methylation panel in an independent cohort

**Figure 3:**
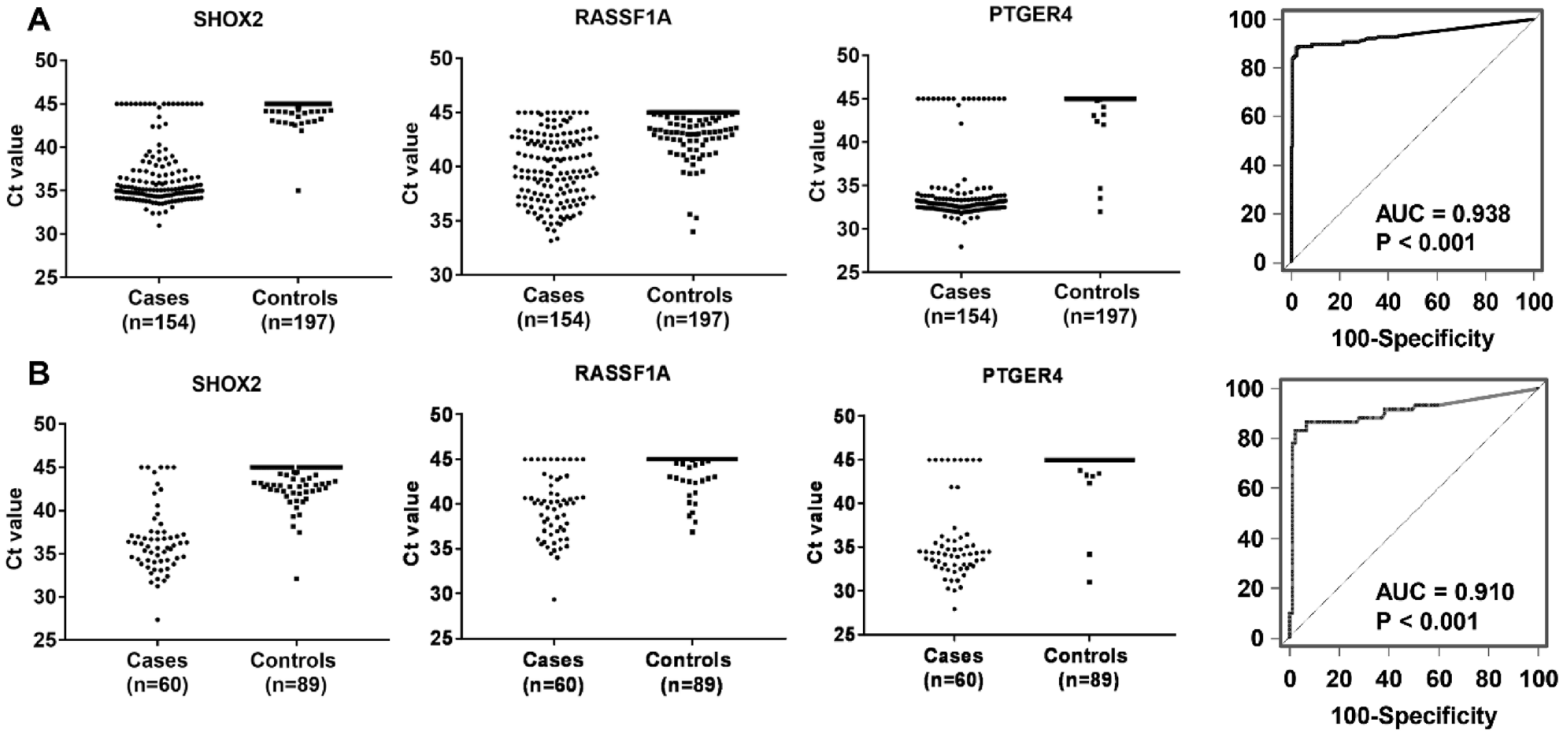
The methylation level distribution of short stature homeobox 2 gene (*SHOX2*), ras association domain family 1A gene (*RASSF1A*), and prostaglandin E receptor 4 gene (*PTGER4*) and diagnostic accuracy (ROC analysis) of the three gene methylation panel in the training cohort (**A**) and validation cohort (**B**).

**Figure 4:**
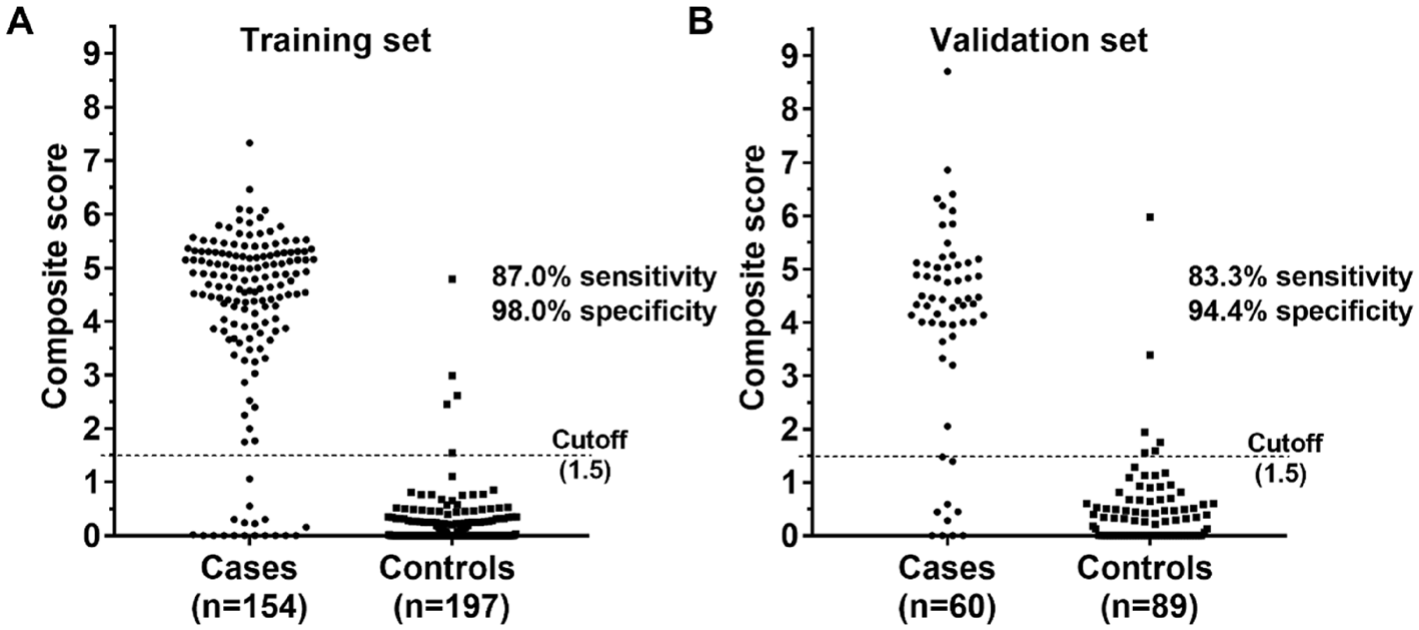
The distribution of the samples’ composite scores in the training and validation set, respectively.

The diagnostic efficiency of the three gene methylation panel was validated in an independent cohort, which comprised 60 LC cases and 89 controls with or without nonmalignant lung disease. The demographic and clinical characteristics of the subjects in the validation set were similar with respect to the training set. The methylation level distribution of *SHOX2*, *RASSF1A,* and *PTGER4* and the results of the ROC analysis for the three gene methylation panel in the validation set were displayed in Fig. 3. The distribution of the three DNA methylation biomarkers was in agreement with the findings observed in the training test, which indicated that the gene methylation could be reproducibly measured. Furthermore, as shown in Fig. 3, the diagnostic performance of the three gene methylation panel in the validation set was close to the training results (AUC = 0.910 versus AUC = 0.938). We used the optimal cut-off (1.5) obtained in the training set to determine the panel’s diagnostic performance in the validated cohort. The panel produced a sensitivity of 83.3% and a specificity of 94.4% (Fig. 4). Taken together, these results confirmed that the three gene methylation panel had the potential for detecting lung cancer.

## Discussion

Low-dose spiral computed tomography (LDCT) can be a reliable screening tool for detecting lung cancer in a high-risk population, which decreases the mortality rate of lung cancer by 20%^5^. However, a severe limitation of LDCT is its low specificity^5,28^, and the rate of surgical resections for the benign disease remains too high (6–38%)^29^. DNA methylation has been shown to play an important role in carcinogenesis at an early stage^30–32^, which makes DNA methylation alterations promising candidates in cancer biomarker research. Besides, methylation assays are relatively easy to set up, perform, and automate. This study demonstrated that the assessment of DNA methylation markers in blood plasma provides a novel noninvasive method to increase lung cancer diagnostic accuracy.

The 3-gene methylation panel in our study demonstrated high discriminatory power to differentiate patients with LC from healthy subjects and patients with nonmalignant diseases of the lung (AUC = 0.938). This panel was validated in a study incorporating patients with various nonmalignant lung diseases such as pulmonary sarcoidosis and bronchiectasis into the control group. The diagnostic efficiency was not influenced by the presence of benign lung disease and other (nonlung) cancers in the control population. In addition, this discrimination is independent of age, gender, and even smoking status, suggesting that it detects cancer-specific alterations rather than tobacco-related field cancerization. The favorable discrimination ability of the panel in our study indicates that it is probably an applicable and feasible method for the detection and diagnosis of LC.

The combined detection of *SHOX2*, *RASSF1A* and *PTGER4* gene methylation in plasma improved detection sensitivity in lung cancer diagnosis compared to previous studies^20,23,33,34^, and achieved high sensitivity across all histological subtypes of lung cancer. Kneip et al. performed DNA methylation analysis of the *SHOX2* gene in blood showed a sensitivity of 60% and specificity of 90% in the diagnosis of lung cancer^20^. Zhang et al. detected methylation changes in 322 subjects with both Sanger sequencing and the Methylated Human *SHOX2* and *RASSF1A* Gene Detection Kit (Tellgen Co. Ltd., Shanghai China). The data showed that the *SHOX2* and *RASSF1A* methylation panel in bronchoalveolar lavage fluid (BALF) yielded a diagnostic sensitivity of 81.0% and specificity of 97.4% with an AUC value of 0.892 in lung cancer diagnosis, but it was showed a lower sensitivity in adenocarcinoma of 69.6%^34^. In our study, the methylation panel achieved high diagnostic with a total sensitivity of 87.0% including all histological subtype groups. In subtype analysis, the sensitivity was above 85% for most histological subtypes, especially in adenocarcinoma, the sensitivity detected of 87.5% was much better than previous studies in bronchoalveolar lavage fluid (BALF) samples^34^.

In addition, the methylation panel achieved a relatively high sensitivity of 82.5% and 90.5% in stage I and stage II patients, respectively, which demonstrated that the panel detection has potential clinical value in the early diagnosis of lung cancer. The exceptional high mortality rate of lung cancer can be attributed to a high degree of late diagnosis^2^. The 5-year survival rate of lung cancer is only 15–19% for all stages. However, outcomes are significantly better in patients diagnosed at an early stage; the 5-year survival rate could increase to 50–60%^2,35^, especially for stage I ranging from 81 to 85%^36^. Thus, it is imperative to detect lung cancer at earlier stages. In our study, the 3-gene methylation panel showed good sensitivity in the early diagnosis of lung cancer, indicating that the panel may be used as an early screening tool for lung cancer, thereby increasing lung cancer survival rates.

However, there exist some limitations to our study. *SHOX2* hypermethylation has been reported to occur frequently in a variety of tumors plasma, including lung cancer, head and neck squamous cell carcinoma (HNSCC), renal cell carcinoma (RCC), and colorectal cancer^37^. We realize, therefore, while the combined detection of *SHOX2*, *RASSF1A*, and *PTGER4* gene in plasma achieved a high detection specificity in lung cancer diagnosis, the exact number of patients in other different tumors may be insufficient; to confirm the results, more clinical data would be needed in further studies. Secondly, the validation of this method is limited to plasma free DNA and EDTA anticoagulant blood collection tubes. Other sample types and blood collection methods have not been performed on the effectiveness. Besides, the free DNA content in plasma samples is low and easily degraded. The samples should be handled and stored strictly according to the requirements; otherwise, it will affect the detection and cause false-negative results.

## Conclusions

The methylation analysis of *SHOX2*, *RASSF1A*, and *PTGER4* panel in plasma showed an efficient diagnostic ability in lung cancer diagnosis. This novel noninvasive test of three biomarkers has potential clinical value. It could be used in stand-alone or in combination with current imaging detection methods to improve the over-all diagnosis of lung cancer.

## Supplementary Information

Supplementary Information.

## Data Availability

The datasets used and analyzed during the current study are available from the corresponding author on reasonable request. Subjects’ data are not publicly available due to them containing information that could compromise participant consent and confidentiality.
