# Supplementary material for: A panel of DNA methylation biomarkers for detection and improving diagnostic efficiency of lung cancer
Source: Sci Rep. 2021 Aug 18;11:16782. doi: 10.1038/s41598-021-96242-6 (PMC8373977; doi:10.1038/s41598-021-96242-6)
Supplement: Supplementary file 1 — Supplementary Information. [file 41598_2021_96242_MOESM1_ESM.docx]

**Supplementary Information**

**A panel of DNA methylation biomarkers for** **detection and improving diagnostic efficiency of lung cancer**

Bing Wei^a,b†^, Fengxin Wu^d†^, Wenqun Xing^c^, Haibo Sun^c^, Chi Yan^a,b^, Chengzhi Zhao^a,b^, Dongqing Wang^a,b^, Xiaobing Chen^c^, Yanli Chen^d^, Mingming Li^d^, Jie Ma^a,b^*

^a^Department of Molecular Pathology, Affiliated Cancer Hospital of Zhengzhou University, Henan Cancer Hospital, Zhengzhou, Henan, China

^b^Henan Key Laboratory of Molecular Pathology, Zhengzhou, Henan, China

^c^Department of Thoracic Surgery, Affiliated Cancer Hospital of Zhengzhou University, Henan Cancer Hospital, Zhengzhou, Henan, China

^d^Excellen Medical Technology Co., Ltd., Beijing, China

†These authors contributed equally to this work.

*** Correspondence:**

Jie Ma, Department of Molecular Pathology, Affiliated Cancer Hospital of Zhengzhou University, Henan Cancer Hospital, No 127, Dongming Road, Zhengzhou, Henan 450008, China

Fax: +86-0371-65587686

Tel: +86-0371-65587686

E-mails: [majie_fzbl@163.com](mailto:majie_fzbl@163.com)

*SHOX2*


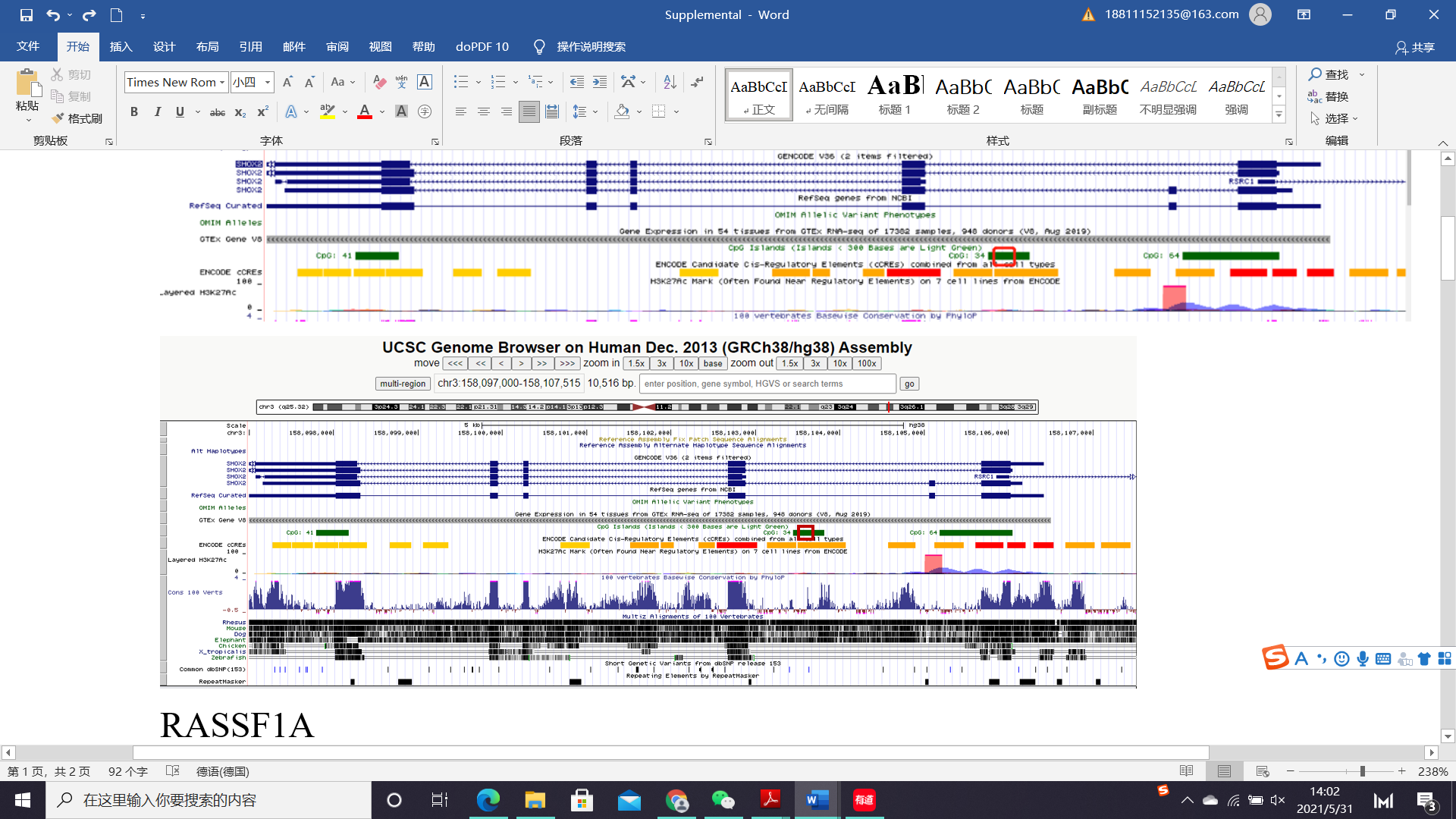


*RASSF1A*


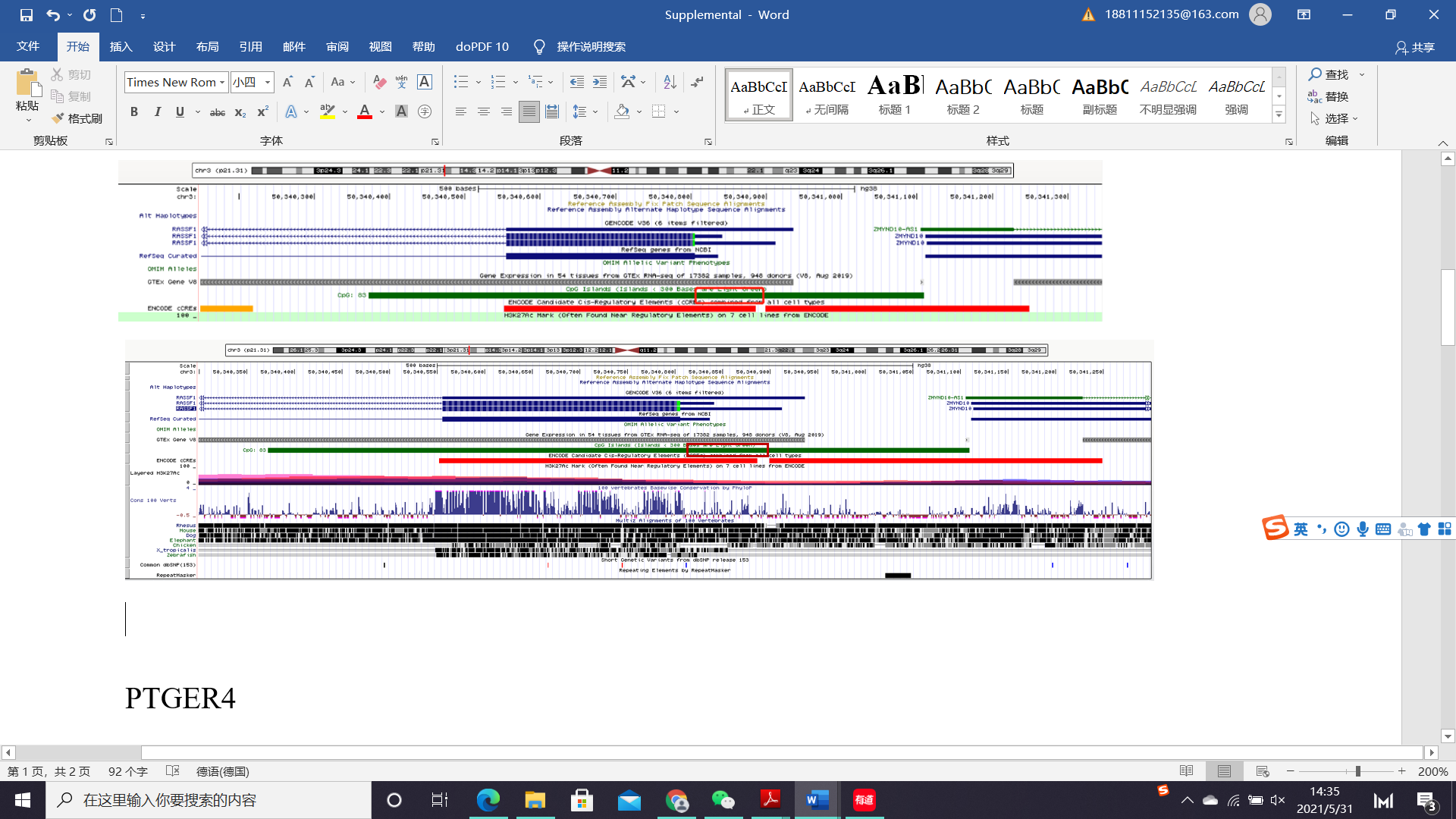


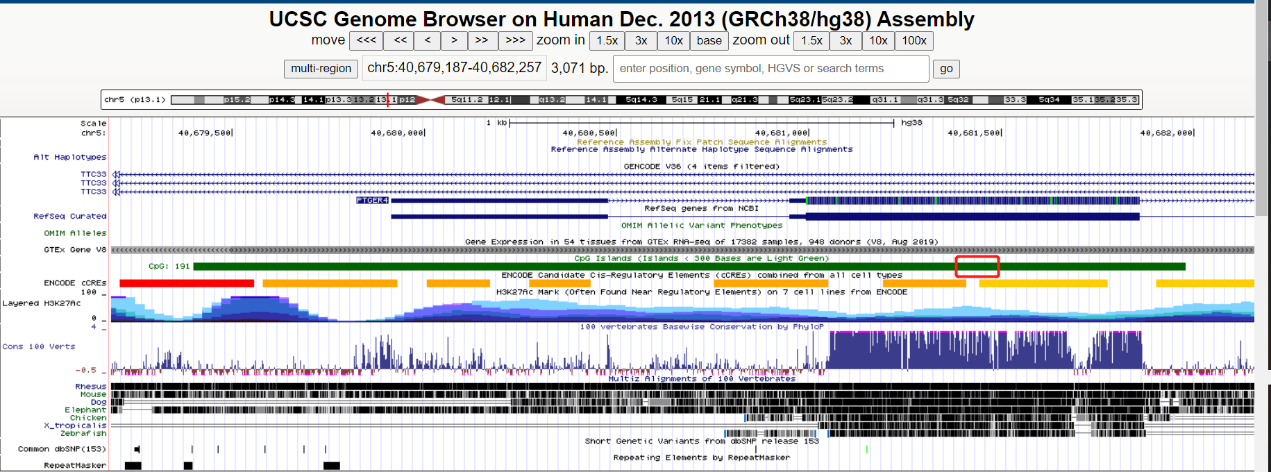
*PTGER4*

**Supplementary Figure S1:** Genomic organization of the *SHOX2,* *RASSF1A*, and *PTGER4* genes and regions targeted for methylation analyses. The qPCR target regions within the genome *SHOX2*: chromosome 3:158,103,514-158,103,606, *RASSF1A*: chromosome 3:50,340,809-50,340,892, and *PTGER4*: chromosome 5:40,681,382-40,681,493 indicated by red boxes. All information was taken from human genome assembly GRCh38/hg38 (http://genome.ucsc.edu).

| **Supplementary Table S1. The primer and the fluorescent probe sequences used in this study.** | | |  |
| --- | --- | --- | --- |
| **Gene** | **Forward primer (5´-3´)** | **Probe (5´-3´)** | **Reverse primer (5´-3´)** |
| ***SHOX2*** | GTTCGTGCGATTTCGGTC | FAM- CAACCAAATAATCTCCGTCCCGC-BHQ1 | TCGCTACCCCTAAACTCGA |
| ***RASSF1A*** | GCGTTGAAGTCGGGGTTCG | JOE-CGCTAACAAACGCGAACCGA-BHQ1 | CCGATTAAACCCGTACTTC |
| ***PTGER4*** | TGGGTATTGTAGTCGCGAGTTATC | Texas Red-CAATCTATACGTCCAACGTACTCTTTTACGCGCTA -BHQ2 | CTACGTAAACAAACGATTAACG |
| ***ACTB*** | GTGATGGAGGAGGTTTAGTAAGT | CY5-ACCACCACCCAACACACAATAACAAACACA-BHQ2 | CCAATAAAACCTACTCCTCCCTT |

**Supplementary Methods**

**Analytical Sensitivity- Limit of Detection**

The analytical sensitivity of the assay was evaluated at four laboratories according to CLSI EP12 and EP17 guidance documents. All sites utilized the test kit manufactured under final manufacturing conditions. Seven levels of technical samples were tested with CpGenome Universal Methylated DNA diluted at concentrations of 0, 8, 16, 24, 30, 40, and 50 pg/ml. The estimated Limit of Detection (LoD) was determined to be 26.83 pg/mL (95% CI 23.86-32.05 pg/mL) using probit analysis.
